# Comparison and evaluation of different methodologies and tests for detection of anti-dsDNA antibodies on 889 Slovenian patients’ and blood donors’ sera

**DOI:** 10.3325/cmj.2011.52.694

**Published:** 2011-12

**Authors:** Polona Žigon, Katja Lakota, Saša Čučnik, Tinka Švec, Aleš Ambrožič, Snežna Sodin-Šemrl, Tanja Kveder

**Affiliations:** University Medical Centre Ljubljana, Department of Rheumatology, Immunology Laboratory, Ljubljana, Slovenia

## Abstract

**Aim:**

To evaluate four different commercially available assays for anti-double stranded DNA (dsDNA) detection and compare them with the in-house radioimmunoassay according to Farr (FARR-RIA) in order to select the optimal primary method for use in combination with FARR-RIA.

**Methods:**

Sera from 583 consecutive patients sent to our laboratory for routine diagnosis, 156 selected patients with autoimmune diseases (76 systemic lupus erythematosus [SLE] patients and 80 patients with other autoimmune diseases), and 150 blood donors were tested for anti-dsDNA antibodies with two enzyme-linked immunoassays (ELISA), two *Crithidia luciliae* immunoflourescence tests (CLIFT), and FARR-RIA. The specificities and sensitivities of the tests were calculated and compared.

**Results:**

FARR-RIA and CLIFT 2 showed the highest specificity for SLE (100%), with CLIFT 2 showing higher sensitivity (33% vs 47%). Both ELISAs showed higher sensitivities (>53%) than FARR-RIA but lower specificities (<93%), whereas CLIFT 1 showed the lowest overall agreement with FARR-RIA.

**Conclusion:**

CLIFT 2 was selected as the primary test for use in combination with FARR-RIA. The use of CLIFT 2 reduced the number of sera that needed to be tested by FARR-RIA, the time needed to report the results, and environmental toxicity, cancerogenicity, and radioactivity.

Anti-double stranded (dsDNA) antibodies were discovered in 1957 and since then have been well recognized as diagnostic markers of systemic lupus erythematosus (SLE). They are excellent indicators of SLE disease activity (1,2) and their elevated levels usually precede exacerbation of disease (sometimes by more than a year) (3). Anti-dsDNA levels rise during flares of SLE disease activity, especially in lupus nephritis (3,4). Many studies questioned the significance of anti-dsDNA antibodies in disease pathology and the association between anti-dsDNA antibodies and disease activity using a variety of different assays (5-9).

Anti-dsDNA antibodies are generally detected and quantified by commercially available kits for enzyme-linked immunosorbant assay (ELISA, also automated versions), *Crithidia luciliae* immunofluorescence assay (CLIFT), and radioimmunoassay methods developed according to Farr technique (FARR-RIA) (9). Different combinations of these methods are used in diagnostic laboratories worldwide, without a consensus on exclusive methods (8,10). An important cause of discrepancies between results obtained with different methods lies in the avidity of antibodies. ELISAs detect antibodies of both low and high avidity, whereas CLIFT and FARR-RIA assays predominantly detect antibodies of high avidity (11). The method of choice in our diagnostic laboratory since the 1970s has been FARR-RIA. This technique was introduced by Wold et al in 1968 (12) and it employs ammonium sulfate precipitation to separate dsDNA/anti-dsDNA complexes from free (radiolabeled) dsDNA. In our assay we use commercially available ^14^C labeled dsDNA from *E. Coli.*

There are certain considerations regarding the detection of anti-dsDNA antibodies that need to be specified. For the diagnosis of SLE, it is crucial that the anti-dsDNA assay is highly specific for dsDNA, especially since elevated levels of anti-dsDNA antibodies can also be detected in other autoimmune diseases, as well as in blood donors, very much depending on the detection method used (13-17). FARR-RIA has the highest specificity for anti-dsDNA antibodies detection but a low sensitivity (18). Therefore, the practical approach has been to use an assay that detects both high and low avidity anti-dsDNA antibodies as a primary screen (19). Such an assay is either CLIFT (20) or anti-dsDNA ELISA (15). When using CLIFT, it is extremely important to score only the kinetoplast fluorescence since nuclei always contain many antigens other than DNA (5). Retesting of positive samples with FARR-RIA not only confirms the diagnosis but also provides the quantitative data allowing the physician to monitor disease activity (8). The problem with anti-dsDNA ELISAs is that they often give false-positive results due to binding of immune complexes (with negatively charged moieties) to the pre-coat intermediates (10,11). An alternative to classic ELISA makes use of biotinylated DNA coating via streptavidin to the plates; however the detection of antibodies against single-stranded DNA remains another deficiency of these tests (19). Antibodies against single-stranded DNA only recognize single-stranded DNA and are specifically directed against purine and pyrimidine bases (21). They are observed not only in patients with SLE but also in other connective tissue diseases, such as systemic sclerosis and myositis (11).

The aim of our study was to evaluate four different commercially available assays for anti-dsDNA detection and compare them to the in-house FARR-RIA assay in sera from 583 consecutively collected individuals, 156 individuals with autoimmune diseases, and 150 blood donors. The overall intent was to substantially shorten the time of reporting results, lower toxicity, and estimate the overall laboratory costs for anti-dsDNA testing.

## Materials and methods

### Participants

This cross-sectional study analyzed 156 sera of Slovene patients with systemic autoimmune diseases. Seventy-six patients (5 men and 71 women, mean age 40.49 ± 11.7) with SLE fulfilled the criteria established by American Rheumatism Association (22) and revised by the American College of Rheumatology (23). The patients’ control group of 80 patients (14 men and 66 women, mean age 51.74 ± 13.6) comprised 16 patients with primary antiphospholipid syndrome (pAPS) who were diagnosed based on the revised International Consensus criteria (24), 35 patients with rheumatoid arthritis, and 29 patients with Sjoegren syndrome. All patients had their sera collected and analyzed at the Department of Rheumatology, University Medical Centre, Ljubljana. In addition, we included consecutive sera of 583 Slovenians that were sent to our laboratory for anti-dsDNA testing from April to July 2009 and the diagnoses/clinical signs of these individuals were unknown to us at the time of the measurements (141 men and 442 women, mean age 46.54 ± 18.8). The donor control group included sera from 150 blood donors (93 men and 57 women, mean age 42.64 ± 11.6). All sera were collected in May 2009, aliquoted, tested by the FARR-RIA in 48 hours and the rest of the sera were stored at -80°C until assayed by ELISAs and CLIFTs. Participants signed an informed consent and the study was approved by the National Medical Ethics Committee, Ljubljana, Slovenia.

### Laboratory measurements

Patient sera were prospectively analyzed with five different anti-dsDNA assays. Four of them were commercially available kits. We used two indirect immunofluorescent tests – NOVA Lite^TM^ dsDNA *Crithidia luciliae* (INOVA Dignostics, San Diego, CA, USA) (CLIFT 1) and Fluorescent nDNA Test system (Immuno Concepts, Sacramento, CA, USA) (CLIFT 2) and two enzyme immunoassays – Diastat^TM^ (Euro-Diagnostica, Malmö, Sweden) (ELISA 1) and Quanta Lite^TM^ dsDNA (INOVA Dignostics) (ELISA 2). All kits were used according to the manufacturer’s instructions. All CLIFT preparations were examined by three biochemical analysts in order to obtain a consensus result. The analysts were blinded to the results of other tests or other clinical information.

The in-house FARR-RIA method used in the Immunology Laboratory since 1976 follows the first published protocol (25) with some adaptations. Briefly, sera complement was inactivated by heating at 56°C for 30 minutes. Five microliters of sera were diluted (1:10) in borate buffer saline (pH = 8.0) in a glass tube and incubated with 100 ng ^14^C dsDNA extracted from *E coli* (Amersham Pharmacia Biotek, Little Chalfont, UK) for 1 hour at 37°C. Samples were stored overnight at 4°C, and the following day saturated ammonium sulfate was added to precipitate proteins (1:1) and incubated for one hour at 4°C. Following a 15-minute-centrifugation at 1800 × g, the supernatants (S) and pellets (P) were divided into separate glass bottles for scintillation counting. Bray scintillation solution was added, and the amount of radiation (cpm counts) was measured in each flask. The ratio (P-S/P+S) above 0.35 was determined as a positive result.

The international reference standard WO/80 was no longer available from the World Health Organization and therefore it was not included in the study.

### Statistical analysis

Statistical analysis was performed using the SPSS 15.0 program (SPSS Inc., Chicago, IL, USA). Correlations of variables were determined by the Spearman rank correlation, and kappa values for agreement were computed. Normality of distribution was evaluated with Kolmogorov-Smirnov test, normal probability plots, and curve fittings. Since data were not normally distributed, differences between the means were analyzed by the Mann-Whitney test. The relative risks were approximated by odds ratios with its 95% confidence interval. The receiver operating characteristic curves (ROC) were constructed, and sensitivity, specificity, and positive and negative predictive values were calculated. A *P* value of <0.05 was considered statistically significant.

## Results

### Normal range and cut-off values for anti-dsDNA assays

The main characteristics of the five anti-dsDNA assays are presented in Table 1. The reference range of the assays was determined by analyzing samples from 150 blood donors. None of the anti-dsDNA results fulfilled the criteria of normal distribution with Kolmogorov-Smirnov test (*P* < 0.001). In ELISA 1, the 99th percentile estimated on our population was on the border between equivocal and positive samples as determined by the manufacturer and in ELISA 2 it was lower than the manufacturer’s, however their calculations included more blood donors.

**Table 1:** Characteristics of the five anti-double stranded DNA (dsDNA) assays*

Assay	Manufacturer	Method	Isotype detection	dsDNA origin	Threshold value between negative and positive†	Slovenian blood donors (n = 150)	
						99th percentile	mean±SD
**ELISA 1**	Euro-Diagnostica	microplate/manual	IgG, IgM	calf thymus dsDNA	50 IU/mL	50.2	18.7
**ELISA 2**	INOVA Dignostics	microplate/manual	IgG	calf thymus dsDNA	300 IU/mL	177.3	87.7
**FARR-RIA**	in-house	RIA/manual	IgG, IgM	[^14^C] DNA extract from *E coli*	0.35	0.11	0.12
**CLIFT 1**	INOVA Dignostics	CLIFT/manual	IgG	kinetoplast DNA	presence of fluorescence at kinetoplast for a serum dilution at 1/10		
**CLIFT 2**	Immuno Concepts	CLIFT/manual	IgG	kinetoplast DNA	presence of fluorescence at kinetoplast for a serum dilution at 1/10		

*Abbreviations: SD – standard deviation; IU – international units; ELISA – enzyme-linked immunoassays; FARR-RIA – radioimmunoassay according to Farr; CLIFT – *Crithidia luciliae* immunoflourescence test.

†According to the manufacturer.

The cut-off value for FARR-RIA anti-dsDNA antibody positivity has been previously determined to be 0.33 (20). Two parameters of frequency distribution (99th percentile and mean +SD) for FARR-RIA results are presented in Table 1. In accordance with our clinical parameters, we set the cut-off value at 0.35 using the diagnostic specificity of 100%. CLIFT 1 found 8 weakly positive and 3 highly positive blood donors, while CLIFT 2 and FARR-RIA found all blood donors to be negative.

### Comparison of four commercially available tests and in-house FARR-RIA

We used 583 fresh sera from persons who underwent anti-dsDNA testing in our Immunology Laboratory. Their diagnoses/clinical signs were unknown to us at the time of the measurements. We determined the levels of anti-dsDNA with four different assays and compared the results with those obtained by FARR-RIA. CLIFT 1 detected the greatest number of positive sera, while FARR-RIA detected the lowest number of positive sera (Table 2).

**Table 2:** Detection of anti-double stranded DNA (dsDNA) antibodies by five different assays in 583 sera sent to Immunology laboratory for routine anti-dsDNA testing*

	No. of patients (cut-off values)^†^				
Assay^‡^	negative	equivocal	weakly positive	moderately positive	highly positive
CLIFT 1	401 (-)	/	79 (+)	99 (++)	4 (+++)
CLIFT 2	513 (-)	/	28 (+)	31 (++)	11 (+++)
FARR-RIA	558 (<0.35)	/	3 (0.35-0.39)	12 (0.40-0.55)	10 (>0.55)
ELISA 1 (IU/mL)	487 (<30)	16 (31-50)	69 (51-300)	/	11 (>300)
ELISA 2 (IU/mL)	500 (<200)	23 (201-300)	44 (301-800)	/	16 (>800)

*Abbreviations: ELISA – enzyme-linked immunoassays; FARR-RIA – radioimmunoassay according to Farr; CLIFT – *Crithidia luciliae* immunoflourescence test.

†The cut-off values, presented in parentheses, were determined according to the manufacturer instructions for CLIFT and ELISA.

There was an overall agreement between CLIFT 2 and ELISA 2 with FARR-RIA of above 90%, and a low agreement between CLIFT 1 and FARR-RIA (73%) (Table 3).

**Table 3:** Correlation of four commercial kits for detection of anti-double stranded DNA (dsDNA) antibodies and the in-house FARR-RIA in 583 sera sent to Immunology laboratory for routine anti-dsDNA testing*

	CLIFT 1		CLIFT 2		ELISA 1		ELISA 2	
	-	+	-	+	-	+	-	+
FARR-RIA								
-	401	158	511	48	499	60	519	40
+	0	24	2	22	4	20	4	20
Spearman coefficient; *P*	0.31; <0.001		0.51; <0.001		0.42; <0.001		0.50; <0.001	
Overall agreement (%)	73		91		89		92	
Kappa^†^	0.173		0.433		0.343		0.443	

*Abbreviations: ELISA – enzyme-linked immunoassays; FARR-RIA – radioimmunoassay according to Farr; CLIFT – *Crithidia luciliae* immunoflourescence test.

†Kappa – Cohen's kappa coefficient, a statistical measure of agreement.

**Figure 1:**
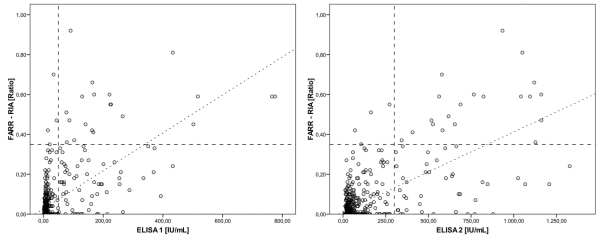
Anti-double stranded DNA antibodies detected with in-house radioimmunoassay according to Farr correlated significantly with results of both enzyme-linked immunoassays, Eurodiagnostica Diastat™ EIA (left panel) and INOVA Quanta Lite™ dsDNA (right panel) in 583 sera sent to Immunology laboratory for routine anti-dsDNA testing.

The levels of anti-dsDNA antibodies detected with both quantitative ELISA anti-dsDNA methods significantly correlated with FARR-RIA (Spearman rank correlation coefficient, (*Rho* = 0.491 – ELISA 1, *Rho* = 0.418 – ELISA 2; *P* < 0.001) (Figure 1).

### Diagnostic accuracy of FARR-RIA as compared with four commercially available anti-dsDNA kits

FARR-RIA and CLIFT 2 showed the highest specificity for SLE (100%) (Table 4). No false positives were determined by either of the assays, which indicates the absence of SLE. CLIFT 2 detected 47.4% of all SLE patients, meaning that it was more sensitive than FARR-RIA. Both ELISAs had higher sensitivity than FARR-RIA when the manufacturers’ cut offs were considered. However, both of them showed a lower diagnostic specificity and consequently lower positive predictive value than FARR-RIA. Setting the cut-off at the 99th percentile estimated on our donors’ control population would even lower the diagnostic specificity of the ELISAs, therefore these calculations were not considered. CLIFT 1 showed the highest sensitivity, but since it had the lowest specificity (with the positive likelihood ratio close to 1), this had little practical significance (Table 4).

**Table 4:** Diagnostic specificity and sensitivity for systemic lupus erythematosus obtained with four different anti- double stranded DNA kits and in-house FARR-RIA in 156 sera of Slovene patients with systemic autoimmune diseases

Assay*		Connective tissue disease^†^	Systemic lupus erythematosus	*P*	Odds ratio	Diagnostic sensitivity (%)	Diagnostic specificity (%)	Positive predictive value (%)	Negative predictive value (%)	Likelihood ratio +	Likelihood ratio -
CLIFT 1	-	35	14	<0.001	3.4	81.6	43.8	57.9	71.4	1.5	0.4
	+	45	62								
CLIFT 2	-	80	40	<0.001	-^‡^	47.4	**100.0**	100.0	66.7	-^‡^	0.5
	+	0	36								
FARR-RIA	-	80	51	<0.001	-^‡^	32.9	**100.0**	100.0	61.1	-^‡^	0.7
	+	0	25								
ELISA 1	-	73	29	<0.001	16.9	61.8	91.3	87.0	71.6	7.1	0.4
	+	7	47								
ELISA 2	-	74	35	<0.001	14.5	53.9	92.5	87.2	67.9	7.2	0.5
	+	6	41								

*Abbreviations: ELISA – enzyme-linked immunoassays; FARR-RIA – radioimmunoassay according to Farr; CLIFT – *Crithidia luciliae* immunoflourescence test.

†Rheumatoid arthritis, Sjoegren syndrome, and primary antiphospholipid syndrome.

‡No false positives detected.

**Figure 2:**
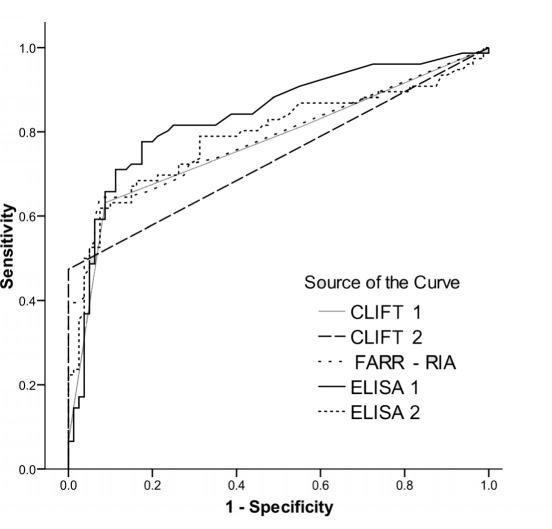
Correlation between specificity and sensitivity of four anti-double stranded DNA kits and in-house radioimmunoassay according to Farr (FARR-RIA). Area under the curve values were the following: *Crithidia luciliae* immunoflourescence test (CLIFT) 1 – 0.771; CLIFT 2 – 0.737; FARR-RIA – 0.787; enzyme-linked immunoassays (ELISA) 1 – 0.835; ELISA 2 – 0.789.

We evaluated the diagnostic applicability of all assays with ROC curve and estimated the area under the curve (Figure 2). The greatest area under the curve was obtained for ELISA 1 and the smallest for both CLIFT assays, partially because they are qualitative assays. FARR-RIA area under the curve was between ELISA and CLIFT, because its cut-off was set for high specificity and consequently lower sensitivity (Figure 2).

**Figure 3:**
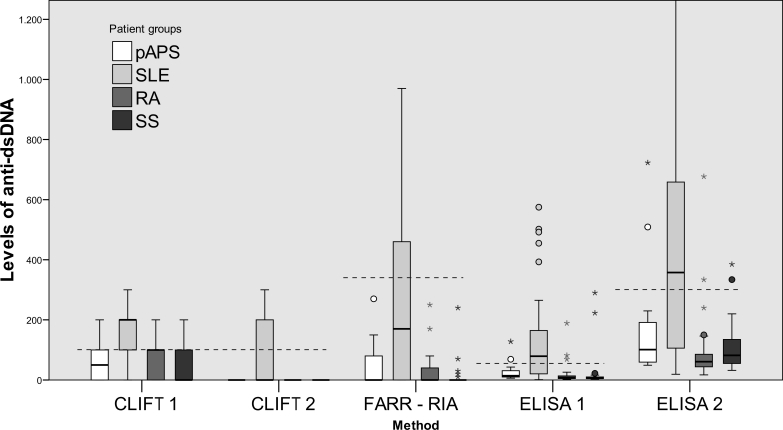
Levels of anti-double stranded DNA (dsDNA) antibodies in patients groups with primary antiphospholipid syndrome (open bars), systemic lupus erythematosus (light gray bars), rheumatoid arthritis (dark gray bars), and Sjoegren syndrome (closed bars) detected by four anti-dsDNA kits and in-house radioimmunoassay according to Farr (FARR-RIA). Dashed lines represent the cut-off value for each individual assay. In order to present all methods in one graph, the results were recalculated based on the following: for *Crithidia luciliae* immunoflourescence test (CLIFT) kits, semiquantitative levels were multiplied by 100, for FARR-RIA the ratio values were multiplied by 1000, and for enzyme-linked immunoassays (ELISA) kit values were unchanged (IU/mL).

CLIFT 2 and FARR-RIA detected only SLE patients as positive for anti-dsDNA. All the other kits also detected patients with pAPS, rheumatoid arthritis, and Sjoegren syndrome (false positives) (Figure 3).

### Overall costs, time of reporting, and toxic/cancerous effects of commercially available tests and FARR RIA

Four tests and FARR-RIA (100%) were compared according to the total costs, time needed for reporting the results, environmental toxicity, and cancerogenous characteristics. FARR-RIA was most cancerogenous and toxic to the environment, had the longest time needed for reporting the results, and was most costly. It also requires special safety measures, training of laboratory personnel, and management of workplace and radioactive waste. CLIFT and ELISA tests showed substantial improvement in all of these categories. Specifically, cost benefits of CLIFT tests and ELISA were respectively 50% and 25% higher than those of FARR-RIA. Also, their time needed for reporting the results after having received the samples was respectively one fifth and one half shorter than that required by FARR-RIA (Table 5).

**Table 5:** Estimation of costs, time of reporting, and toxic/cancerogenous chemicals for each anti-double stranded DNA test

Assay*	Overall costs (% of FARR-RIA)†	Time for reporting (% of FARR-RIA)^‡^	Toxic/cancerogenous chemicals^§^
CLIFT 1	50	20	0.09% NaN_3_ as a preservative
CLIFT 2	50	20	0.09% NaN_3_ as a preservative
ELISA 1	75	50	0.5% NaN_3_ in wash buffer and sample diluents, phenolphthalein monophosphate, bronidox L, diethanolamin in substrate, NaOH in stop solution
ELISA 2	75	50	0.5% NaN_3_ as preservative, 0.02% chloramphenicol in sample diluents, poisonous/corrosive chemical in conjugate, irritant in substrate, H_2_SO_4_ in stop solution
FARR-RIA^║^	100	100	^14^C labeled DNA, Bray scintillation fluid: 2.5-diphenyloxazole, naphthalene, methanol, dioxan, ethylene glycol

*Abbreviations: ELISA – enzyme-linked immunoassays; FARR-RIA – radioimmunoassay according to Farr; CLIFT – *Crithidia luciliae* immunoflourescence test.

†Personnel, reagent, and laboratory maintenance costs.

‡Time between the arrival of the sample in the laboratory and reporting the result back to the clinicians.

§The qualitative description of all toxic agents used in the particular anti-dsDNA test.

║The arbitrary scoring scale was set at 100 for FARR-RIA and all other tests were compared to FARR-RIA in percentages.

## Discussion

We determined that CLIFT 2 was the most suitable method for anti-dsDNA detection in combination with FARR-RIA. Both CLIFT tests showed higher sensitivity than FARR-RIA and CLIFT 2 showed a higher specificity and a much higher overall agreement with FARR-RIA without losing any truly positive samples. However, CLIFT as a qualitative assay cannot substitute FARR-RIA, but can reduce its use by approximately 80%, since the results of 583 consecutive sera showed that only every 10th to 15th patient was tested positive for anti-dsDNA by CLIFT and needed quantitative confirmation by FARR-RIA.

For both ELISAs, as qualitative assays, we determined the reference range and cut-off values in our population of blood donors. Their diagnostic specificities were lower than those of FARR-RIA. Our calculations of 99th percentile set the cut-off values lower than the manufacturers’ and therefore reduced the specificity even further. Including either ELISA in our routine practice would increase the number of false-positive results, which would need to be further tested with a more specific assay. In this way, our aim of reducing the number of tests performed with FARR-RIA, while not missing any truly positive samples, would not be achieved. So, ELISAs are not used in our laboratory to detect anti-dsDNA antibodies.

Recently, a similar study (18) compared FARR-RIA with 3 commercial ELISAs and CLIFT. Their conclusion also confirmed our findings that the FARR-RIA assay performed better than other assays and was the best assay to distinguish between patients with quiescent to mildly active SLE and patients with a more active type of SLE.

Having compared CLIFT, ELISA, and RIA, and considered both sensitivity and specificity, we conclude that greater sensitivity does not guarantee a larger number of truly positive samples. The major problem of anti-dsDNA ELISAs represents nonspecific binding to the, plastic surface. However, their availability, ease of use, and quantitative output has kept ELISAs as methods of choice in many laboratories (26). An alternative to anti-dsDNA ELISAs and precoat problems are assays using biotinylated DNA and its streptavidin detection. This diminishes a lot of nonspecific binding, as pointed out by Isenberg and Smeenk (27). The same authors stress the problems of different methodologies, especially different sources of antigen, presentation of the antigen to the antibodies, as well as conditions of assay procedures. New generations of automated enzyme immunoassays (multiplex assays using the Luminex 100 system consisting of distinct uniform 5.5 µm color-coded microspheres) and the latest assays (protein microarrays to detect immunologic targets) are now available, however they still need optimizations and extensive evaluation with FARR-RIA, CLIFT, and ELISA (10,27). In 2010, Antico et al (28) reported on new-generation immunoassays as an effective alternative to FARR-RIA technique and CLIFT. They examined 5 different tests: chemiluminescent immunoassay, fluorometric enzyme immunoassay, two classical ELISAs, CLIFT, and FARR. The conclusion was that, although tested on a limited number of samples, modern enzyme immunoassays for detecting anti-dsDNA antibodies represented a valid alternative to FARR (28), and CLIFT could be used as a confirmatory test in enzyme immunoassay-positive sera.

There were also reports on a novel surface plasmon resonance biosensor chip analytic method (29) and an innovative quantitative electrochemical detection of anti-DNA antibodies (30). Just recently, a novel method using electrophoretic mobility shift assay was reported by Keyhani et al (31), which showed in vivo and in vitro detections of a complex formation between plasma membrane DNA and IgG from SLE patients. However, more studies on larger sample sizes need to be performed before these novel methods are introduced into routine practice.

Our study confirmed that we can decrease the use of FARR-RIA with the introduction of CLIFT 2 assay and obtain the highest possible specificity and sensitivity. Since our original intent was not to choose a screening test, but rather to perform a lower number of FARR-RIA tests, CLIFT 2 proved to be the optimal. Thus, considerably fewer sera need to be tested by FARR-RIA following the use of CLIFT 2 in order to quantify values and perform disease activity monitoring. In this way, we also decreased the time needed for reporting negative results, the amount of toxic, cancerogenous, and radioactive chemicals in the environment, and overall laboratory costs for anti-dsDNA testing. On the basis of all this, we recommend the use of CLIFT 2 followed by FARR-RIA on positive samples, as well as finding a nonradioactive alternative to FARR-RIA.
